# Gas6/TAM Axis Involvement in Modulating Inflammation and Fibrosis in COVID-19 Patients

**DOI:** 10.3390/ijms24020951

**Published:** 2023-01-04

**Authors:** Manuela Rizzi, Stelvio Tonello, Davide D’Onghia, Pier Paolo Sainaghi

**Affiliations:** Department of Translational Medicine, Università del Piemonte Orientale (UPO), 28100 Novara, Italy

**Keywords:** Gas6, TAM receptors, inflammation, fibrosis, COVID-19

## Abstract

Gas6 (growth arrest-specific gene 6) is a widely expressed vitamin K-dependent protein that is involved in many biological processes such as homeostatic regulation, inflammation and repair/fibrotic processes. It is known that it is the main ligand of TAMs, a tyrosine kinase receptor family of three members, namely MerTK, Tyro-3 and Axl, for which it displays the highest affinity. Gas6/TAM axis activation is known to be involved in modulating inflammatory responses as well as fibrotic evolution in many different pathological conditions. Due to the rapidly evolving COVID-19 pandemic, this review will focus on Gas6/TAM axis activation in SARS-CoV-2 infection, where de-regulated inflammatory responses and fibrosis represent a relevant feature of severe disease manifestation. Furthermore, this review will highlight the most recent scientific evidence supporting an unsuspected role of Axl as a SARS-CoV-2 infection driver, and the potential therapeutic advantages of the use of existing Axl inhibitors in COVID-19 management. From a physiological point of view, the Gas6/TAM axis plays a dual role, fostering the tissue repair processes or leading to organ damage and loss of function, depending on the prevalence of its anti-inflammatory or profibrotic properties. This review makes a strong case for further research focusing on the Gas6/TAM axis as a pharmacological target to manage different disease conditions, such as chronic fibrosis or COVID-19.

## 1. Physiological Role of the GAS6/TAM Axis: An Overview

Gas6 (growth arrest-specific gene 6) is a secreted 75 kDa glycoprotein, whose function is vitamin K-dependent, and is expressed in many cell types and tissues, such as those of the heart, lungs, stomach, kidney, pancreas, bone marrow, central nervous system, and gut [1,2,3,4,5,6,7,8]. In physiological conditions, plasma Gas6 levels are around 18–50 ng/mL, which are sufficient to ensure its homeostatic functions [4,9]. Gas6 is known to be involved in regulating many biological processes, ranging from cell proliferation, adhesion and migration, to efferocytosis and apoptosis. Furthermore, its biological functions have been associated with the regulation of platelet activation, inflammatory responses and repair/fibrotic processes [3,10,11,12,13].

The Gas6 structure is characterized by the presence of γ-carboxylated domains and of a sex hormone-binding globulin (SHBG) domain which are essential for its bioactivity. In particular, the γ-carboxylated domains account for the ability of Gas6 to bind phosphatidylserine (PtdSer) residues, while the C-terminal SHBG domain accounts for the interaction of Gas6 with its specific receptors [6,14,15,16,17].

To exert its biological activities, Gas6 needs to interact with a specific tyrosine kinase receptor family, collectively named TAM, consisting of three different members, namely Tyro-3, Axl and MerTK, that can be isolated in human plasma as soluble decoy receptors (sTAM: sTyro-3, sAxl, and sMerTK), generated after cleavage of the transmembrane full-length form by membrane matrix metalloproteinases ADAM-10 and ADAM-17. The formation of such Gas6/sTAM complexes represents a useful protection mechanism, intended to limit ligand-mediated signaling and, thus, to prevent the accidental activation of downstream TAM signaling pathways in cells and tissues [14,15,18,19,20,21].

It is noteworthy that Gas6 shows different binding affinities to the different members of the TAM family: it binds with the strongest affinity to Axl (with a Kd in the nM range), and with the lowest affinity to MerTK, with a Kd in the µM range [22,23]. Despite the different binding affinities to TAM receptors, the downstream activation mechanism is similar: once the ligand is bound, the receptor dimerizes and its tyrosine kinase domains become activated, mediating various cellular responses, such as cell growth and proliferation, the regulation of apoptosis, as well as the modulation of vascular and inflammatory responses in a cell and tissue-dependent manner [4,15,18,20,24]. The different cellular responses triggered by the Gas6/TAM axis activation mainly rely on well-known intracellular signaling mediators such as the p38/MAPK, the PI3K/Akt, the ERK1/2 and the JAK/STAT pathways, which need to work in a coordinated manner to ensure homeostasis in the whole organism [25,26,27].

Due to the rapidly evolving COVID-19 pandemic, Gas6/TAM axis activation has gained interest due to its involvement in inflammatory responses and subsequent fibrosis, two features strictly related to severe SARS-CoV-2 clinical manifestations [28].

## 2. Gas6/TAM Axis Role in Modulating Inflammation

TAM receptors and their ligands were first described as components of innate immunity, but it is now evident that they function at the interface of innate and adaptive immunity. Nowadays, it is known that the Gas6/TAM axis plays a key role in regulating immune homeostasis by sustaining negative feedback-mediated toll-like receptor (TLR) signaling. When the specific ligand binds to TLR, the levels of proinflammatory mediators rise to counteract the noxious stimuli; thus, to avoid extensive damage due to hyperinflammation, cellular protection mechanisms are needed. The Gas6/TAM signaling pathway is the mechanism used by antigen-presenting cells to limit the over-activation of the inflammatory response, by facilitating apoptotic cell recognition by phagocytic cells and by modulating the associated proinflammatory response. To switch off the inflammatory response, apoptosis is a key step: dying cells expose on the outer leaflet of their membrane PtdSer residues, acting as “eat-me signals” for macrophages. Gas6 has been shown to be able to bind such phospholipidic residues, acting as a bridging molecule and allowing the interaction with TAM receptors on phagocyte membranes [29,30]. The resulting apoptotic cell death reduces proinflammatory cytokine production (i.e., IL-6 and TNFα) while inducing the release of anti-inflammatory mediators and the development of immune tolerance. Furthermore, TAM receptors have been described to be present on activated T regulatory cells, thus further supporting the Gas6 anti-inflammatory role [14,15,17,20,31].

**Figure 1:**
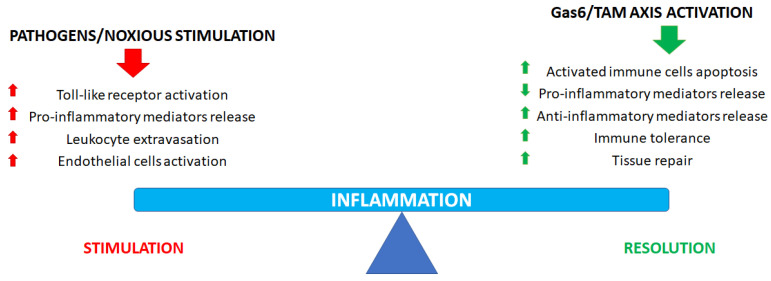
Role of the Gas6/TAM axis in inflammatory process regulation and resolution.

Accumulating evidence supports the view that the Gas6/TAM axis activation could show anti-inflammatory properties in certain cells and tissues while sustaining proinflammatory responses in others (Figure 1), where it supports leukocyte extravasation, endothelium activation and even graft rejection. In the context of inflammation, this dual action is particularly evident in sepsis: indeed, in this pathological condition, it is known that plasma Gas6 levels show a sustained increase, which is correlated to disease severity [4,10,11,32]. Gas6/TAM involvement in proinflammatory responses is also evident in thrombosis, a pathological manifestation showing a strong interdependence with inflammation in several clinical conditions [33]. To date, scientific evidence is available to confirm that Gas6/TAM signaling pathway activation is essential for full and sustained platelet activation and thrombus stabilization, as well as to sustain the development of a prothrombotic milieu, as confirmed by animal studies showing that Gas6 or single TAM receptor knockout mice are less prone to thrombosis than wild-type mice [1,4,7,34,35].

Finally, it is noteworthy that the Gas6/TAM axis is involved not only in immunomodulation but also in promoting tissue repair after injury (Figure 1). When tissue damage occurs, an early inflammatory response is essential to avoid pathogen contamination and to promote tissue remodeling. Such a role is of particular importance when a vessel is damaged when TAM signaling is essential to promote hemostasis to halt bleeding: TAM receptors and ligands stabilize platelet aggregates, promote endothelial cell survival and, finally, the restoration of the physiological endothelial barrier function [4,24].

## 3. Gas6/TAM Axis Role in Modulating Fibrotic Evolution

When a tissue is damaged, the first host response is represented by inflammation, which is followed, in many cases, by tissue healing with extracellular matrix deposition; however, the repair process may become excessive and even harmful in some cases with a fibrotic response. The Gas6/TAM axis has also been shown to be involved in this biological event, where it supports the deposition and remodeling of the extracellular matrix. A fine and timely regulation of fibrotic response is necessary to support physiological wound healing: as discussed for inflammation, also in the case of fibrosis, a de-regulated response could result in a negative clinical evolution, with diffuse fibrosis impairing the physiological activities of the involved tissues. In particular, fibrosis is triggered by virtually all types of insults and affects all organs and tissues [28,36]. Moreover, in virtually all progressive chronic diseases, fibrosis becomes irreversible over time, thus accounting for a progressive loss of functionality of the involved tissue [37,38].

Gas6/TAM signaling pathway involvement has been associated with fibrotic evolution in different body districts, such as the lungs, kidneys, intestines, or liver [39,40,41].

Historically, attention has been focused on the Gas6/TAM axis involvement in liver diseases, highlighting its protective role in response to liver injury [40,42]. Studies on animal models have demonstrated an increase in Gas6 levels after liver injury: in this context, its protective role is directed to the key matrix-producing cellular types and mainly depends on the antiapoptotic effect for hepatic stellate cells and the activating effect toward myofibroblasts [43,44,45,46]. According to the animal data, Gas6 and sAxl have been proposed as biomarkers for chronic liver disease: according to these observations, such biomarker expressions differently change between patients with established/advanced liver fibrosis and patients with initial or no fibrosis, thus supporting that the Gas6/sAxl axis exerts a profibrotic role in chronic liver diseases [43,47,48].

Another district where Gas6/TAM-driven fibrotic evolution has been studied is in the lungs, where inflammation and subsequent fibrosis are key pathogenic features of interstitial lung diseases. Also in this context, studies on cellular and animal models have shown the involvement of this signaling pathway in inflammation and fibrosis modulation [49,50,51,52,53,54]. In vitro and in vivo data were also confirmed by the analysis of Gas6/TAM pathway activation in patients suffering from idiopathic pulmonary fibrosis (IPF), a disease characterized by lung parenchyma deterioration where an elevated Gas6/Axl expression, along with an elevated Axl phosphorylation, could be observed in lung tissues [52,55].

According to the observed involvement of the Axl signaling pathway in different experimental fibrosis models, in recent years, its pharmacological inhibition has been proposed as a new therapeutic option. In particular, a promising candidate is represented by bemcentinib (also known as BGB324 or R428), an Axl inhibitor already tested in solid cancers. This small molecule showed the ability to greatly reduce liver fibrosis and inflammation in a mouse model of NASH (nonalcoholic steatohepatitis) [56] or of CCl_4_-induced damage [43], as well as to mitigate mitochondrial dysfunction and renal fibrosis in a unilateral ureter obstruction murine model [41,57] and to ameliorate pulmonary fibrosis in the humanized SCID/Bg mice model [52]. Furthermore, bemcentinib-mediated Axl signaling inhibition has also been studied both in vitro and in vivo in the context of Crohn’s disease, a pathological condition characterized by fibrotic evolution after chronic inflammation. In such experimental models, bemcentinib treatment resulted in the abrogation of matrix stiffness and TGF-β-induced fibrotic process, along with the sensitization of intestinal myofibroblasts, the main drivers of fibrosis in this organ, to apoptosis [39].

## 4. Gas6/TAM Axis in COVID-19

Due to its immunomodulatory role, as well as its involvement in the modulation of inflammation and subsequent fibrotic evolution, the Gas6/TAM axis is emerging as an interesting research item in the context of the ongoing COVID-19 pandemic, caused by SARS-CoV-2. This new viral agent is a positive, enveloped, single-stranded RNA virus, with high genetic similarity with both SARS-CoV and MERS-CoV, two epidemic coronaviruses responsible for two other severe pneumonia outbreaks in 2002 and 2012, respectively [58,59,60].

SARS-CoV-2-positive patients show different clinical manifestations, ranging from asymptomatic forms or mild flu-like presentation to severe interstitial pneumonia, acute respiratory distress syndrome (ARDS) and severe multiorgan failure, leading, in the most severe cases, to death [58,61,62,63,64,65]. A distinctive hallmark of severe COVID-19 manifestations is the aberrant immune response following pathogen recognition, leading to uncontrolled production and release of proinflammatory mediators, accounting for the so-called “cytokine storm”, which appears to have peculiar characteristics in COVID-19 compared to what is observed in other non-COVID-19-related manifestations [66,67]. Such hyperinflammatory response correlates with disease severity and is characterized by an increase in proinflammatory cytokines, both in the bloodstream and in the bronco–alveolar lavage fluids [61,65,66,67,68,69].

Many studies have reported that a great percentage of patients surviving COVID-19 infection still display respiratory impairment even after discharge, resulting in a reduction in some key physiological parameters such as total lung capacity, forced vital capacity and forced expiratory volume as well as gas transfer ability, finally resulting in a long-term progressive and irreversible deterioration of lung function [61,62,70,71,72,73,74,75].

Consistently, recent studies showed that a large proportion of severe COVID-19 survivors develop fibrotic changes in the lung persisting for months after discharge, especially in elderly, male and mechanically ventilated patients, displaying high levels of inflammation markers (i.e., C-reactive protein (CRP), IL-6, lactate dehydrogenase (LDH), D-dimer). Furthermore, it has been observed that the degree of inflammation and the extent of lung tissue damage correlate with the degree of lung fibrosis, supporting the observed high prevalence of such complication in the most critical patients compared to those experiencing only a mild or moderate form of COVID-19 [70,72,75,76].

Considering its effect in modulating host immune responses, the Gas6/TAM axis has also gained attention in the context of COVID-19 studies, showing a direct correlation between plasma Gas6 levels and disease severity [77,78,79,80]. Table 1 and Table 2 summarize the most relevant papers investigating Gas6 and TAM receptor involvement in SARS-CoV-2 infection and subsequent disease.

The first work theorizing a correlation between the Gas6/TAM axis and COVID-19 was a review by Tutusaus and colleagues, who suggested the TAM pathway involvement at different stages of SARS-CoV-2 infection, mainly focusing on viral mimicry and immunothrombosis, which is often observed as a complication in severe patients experiencing ARDS [92]. Since the publication of that work, many research groups focused their attention on this signaling pathway activation in COVID-19 to disclose the existing correlations between the Gas6/TAM axis and disease evolution. To date, most of the available studies in the literature on this topic date back to the first wave of the pandemic and show some important limits. The most significant limitations of these studies are represented by the wide difference in disease severity at admission and the hospital management of patients, as no clear therapeutic guidelines were available at that time, so, in many cases, information about pharmacological treatment is missing in published reports.

In their study, Morales and coworkers evaluated plasma Gas6 and sTAM expression at admission to emergency care units and observed a direct correlation between basal Gas6 and sAxl levels and disease severity [80]. Similar results were obtained also by Huckriede and colleagues, who studied a cohort of patients admitted to the ICU with severe disease, observing that plasma Gas6 levels were significantly higher in nonsurvivors compared to patients recovering from the disease, allowing good discrimination of patients who will develop irreversible acute lung injury. On the other hand, they did not find any correlation between sAxl levels and organ damage, further highlighting the importance of Gas6 in predicting disease evolution [79]. De Bruin’s research group also obtained similar results in a cohort of patients admitted to the ICU and general wards, where a correlation between plasma Gas6 levels and negative disease evolution was observed [78].

Recently, our group [77] confirmed the correlation between Gas6 levels at the time of hospital admission and an adverse outcome in hospitalized COVID-19 patients. The key feature of this study is represented by its timing and design: patients’ enrollment took place during the third pandemic wave (from January to May 2021) and, most importantly, all the enrolled patients received standard pharmacological therapy (corticosteroids and low-molecular-weight heparin) according to the hospital guidelines for the management of SARS-CoV-2-positive patients. Furthermore, enrollment was restricted only to hospitalized patients presenting moderate to severe respiratory failure and needing noninvasive ventilation. These are other added values compared to the previous studies on this topic as they guarantee a homogeneous study cohort. Furthermore, the results obtained in this study highlighted a decrease, even if not statistically significant, of Gas6 plasma levels over time [77], thus supporting the assumption that this molecule behaves as an acute-phase molecule [10,11], playing an important role in COVID-19-related hypercytokinemia and the altered coagulation state, typical of severe disease conditions [96,97].

It is important to note that almost all reports in the literature about the involvement of the Gas6/TAM axis in COVID-19 focus on the adult population. To date, only one study [81] evaluated this issue in pediatric patients, highlighting that, in contrast to what was observed in adults, both Gas6 and MerTK levels are lower in infected individuals than in healthy individuals, an observation that further supports the different disease evolution according to the age of the infected patients.

## 5. Axl Role in SARS-CoV-2 Infection

Even if the majority of the research papers are focused on the Gas6/TAM axis involvement in COVID-19, interesting results also come from in vitro research. Since the first decade of this century, different studies demonstrated, in vitro, that TAM receptors and their ligands, by acting as a bridge with PtdSer, could promote different enveloped virus infections (i.e., filovirus such as Ebola, and flaviviruses such as Dengue and West Nile) [22,25,92,98]. In particular, the Axl role in lung viral infections has also been studied in a murine model, where it has been demonstrated that its inhibition by monoclonal antibodies locally enhanced innate and adaptive immunity, suggesting Axl-targeted inhibition as an interesting clinical approach to treat viral lung diseases [99]. It is noteworthy that, according to available in vitro and in vivo evidence, Axl is not indispensable for enveloped virus entry, but might reasonably act as a “facilitator” in some cell types rather than others [22].

According to such evidence, Axl has also been investigated in the context of COVID-19 and in vitro results have highlighted an unsuspected role of Axl in the SARS-CoV-2 infection process, even if its exact mode of action has not yet been clarified. In particular, some reports demonstrated that the Axl receptor can specifically interact with the N-terminal domain of SARS-CoV-2 spike protein in an ACE2-independent manner, thus representing a potential alternative receptor for viral entry in pulmonary and bronchial epithelial cells, where Axl and ACE2 receptors are not co-expressed. These in vitro studies highlighted that knocking down Axl or its addition in the soluble recombinant form to cell culture is effective in reducing the viral infection of pulmonary epithelial cells, while its biological ligands (Gas6 and protein S) do not bind to SARS-CoV-2 [82,93]. According to this evidence, Axl may be involved in the viral endocytosis mechanism by interacting with virion-associated PtdSer residues [83,94]. Consistently, the inhibition of the intracellular Axl signaling pathway with bemcentinib reduced receptor-mediated viral internalization and new virions production in a dose-dependent manner [83].

This ability of SARS-CoV-2 to exploit different cellular receptors to infect host cells not only offers a reasonable explanation for its high infectivity and its wide tropism but also represents a new therapeutic target to limit COVID-19 spreading. Although Axl’s involvement in tumor progression has been known for many years, different drugs targeting this receptor have already been developed and commercialized, fostering studies about their repurposing in COVID-19 management. As preclinical studies using Axl inhibitors such as bemcentinib and gilteritinib showed promising results [84,85,86,87,95], bemcentinib is under clinical trials to evaluate its effectiveness in treating SARS-CoV-2 infection [88,89], while it has been reported that gilteritinib administration in an acute myeloid leukemia patient ameliorated COVID-19 symptoms [90].

In addition to these studies investigating Axl’s role as an alternative receptor for SARS-CoV-2 cell entry, a recent study focused on Axl involvement in COVID-19 pathogenesis, especially in the epithelial-to-mesenchymal transition (EMT) process [91]. The SARS-CoV-2 infection has been shown to upregulate different oncogenic pathways, including EMT [91,100]. Such an alteration in the adhesive properties of epithelial cells, especially in the lung district could thus be involved in altering air/blood barrier permeability, finally resulting in impaired respiratory function, typical of severe COVID-19. Considering Axl’s role in regulating EMT, Stewart and coworkers hypothesized that reverting EMT using Axl inhibitors such as bemcentinib, which displays a proven in vitro antiviral efficacy against SARS-CoV-2, could represent an attractive option to limit COVID-19 severity [91].

## 6. Conclusions

In recent years, many research groups have focused attention on the Gas6/TAM axis involvement in inflammation and fibrotic evolution in the context of different human diseases.

To date, it is known that in acute tissue damage settings, Gas6/TAM signaling plays a protective role based on its anti-inflammatory properties and its profibrotic abilities, finally sustaining the repair process. On the other hand, in many chronic diseases, this effect turns into a negative one, i.e., when chronic tissue damage occurs, Gas6/TAM activity balance shifts toward the profibrotic side, accounting for an abundant extracellular matrix deposition that progressively leads to organ parenchymal damage and loss of function.

Information about Gas6/TAM’s physiologic role is still evolving and, in the last few years, such signaling pathways have been identified as a prognostic biomarker for COVID-19 evolution. Indeed, in SARS-CoV-2-positive patients, Gas6 behaves as an acute-phase protein, and its increase in plasma correlates with a negative prognosis. This last observation could be related to the ability of Gas6 to bind to PtdSer residues, which is emerging as an interesting feature in viral infection. In this case, Gas6 is thought to act as a bridging molecule between viral envelope PtdSer residues and host TAM receptors, which therefore act as virus entry factors, enhancing viral tropism through the so-called “apoptotic mimicry” mechanism [15,16,101,102,103,104].

Based on the most recent evidence about the Gas6/TAM axis involvement in COVID-19 severity and considering its well-recognized profibrotic role, such a signaling pathway appears of great clinical interest to not only support the early stratification of patients but also to predict long-term disease sequelae, such as lung fibrosis. Despite the existing evidence of the reliability of Gas6 as a COVID-19 negative disease evolution marker, to date, investigations about its predictive role for long-term lung fibrosis development in survivors are still lacking. Considering ongoing evidence regarding the high number of survivors showing lung fibrosis persisting for months after discharge, it would be of great interest to evaluate the Gas6/TAM involvement in such progressive and irreversible disease sequelae, to promptly establish the appropriate pharmacological therapy, thus increasing survivors’ quality of life.

Due to the limited number of studies published on the Gas6/TAM system involvement in COVID-19 pathogenesis, further studies are warranted to better elucidate the role of this pathway in this disease.

In conclusion, there is accumulating evidence of Gas6/TAM pleiotropic physiological roles, thus fostering future research studies focusing on identifying safe and effective clinical interventions targeting this signaling pathway to manage different disease conditions such as chronic fibrosis or the current COVID-19 outbreak.

## Figures and Tables

**Table 1:** Summary of the most relevant literature (in vitro and clinical studies, clinical trials, and case reports) regarding Gas6 and TAM receptors involvement in SARS-CoV-2 infection and COVID-19 progression and management.

Article Type	Main Findings	Reference
Clinical study	In a cohort of moderate/severe COVID-19 patients admitted to the high-dependency/subintensive ward during the third wave of the pandemic, plasma Gas6 levels at admission predicted an adverse disease outcome.	[77]
Clinical study	In a cohort of COVID-19 patients admitted to the general wards or the intensive care unit during the first wave of the pandemic, plasma Gas6 levels correlated with negative disease evolution.	[78]
Clinical study	In a cohort of severe COVID-19 patients admitted to the intensive care unit during the first wave of the pandemic, plasma Gas6 levels discriminated survivors from nonsurvivors.	[79]
Clinical study	In a cohort of COVID-19 patients admitted to the emergency department during the first wave of the pandemic plasma Gas6 and Axl levels reflect COVID-19 severity and could predict disease evolution.	[80]
Clinical study	In a cohort of COVID-19 patients admitted to the pediatric emergency department, plasma Gas6 and MerTK levels were lower when compared to healthy controls.	[81]
In vitro study	Identification of Axl as a candidate receptor involved in SARS-CoV-2 infection and as a potential pharmacological target for clinical interventions. SARS-CoV-2 spike protein has been described as able to bind the Axl receptor and to use it as an alternative entry route, as confirmed by the lower viral load observed after Axl knockout or blocking with the soluble recombinant protein. Based on such observations, the authors suggest the use of soluble recombinant human-grade Axl as a potential therapeutic intervention in COVID-19 patients.	[82]
In vitro study	Overview of Axl’s role in SARS-CoV-2 infection and role of its inhibitor bemcentinib as an antiviral agent. SARS-CoV-2 spike protein has been described as able to directly bind Axl, which can then act as an alternative receptor for virus entry, and the pharmacological inhibition of the Axl pathway by bemcentinib strongly reduced viral load.	[83]
In vitro study	Identification of gilteritinib as an antiviral agent against SARS-CoV-2. Gilteritinib’s antiviral effect is supposed to rely on its ability to activate innate immunity by blocking Axl, which acts as an inhibitor of innate immune responses.	[84]
Preclinical study	Identification of gilteritinib as an in vitro antiviral agent and confirmation of its protective effect in vivo (Syrian hamster model). Gilteritinib’s antiviral effect has been supposed to rely on its ability to interfere with Axl-mediated viral entry.	[85]
In vitro study	Identification of bemcentinib as an antiviral agent against SARS-CoV-2 in different cellular lines. The authors suppose that the observed pharmacological effect relies on Axl involvement in viral entry, as previously observed for other viral agents.	[86]
In vitro study	Identification of gilteritinib as a potent antiviral agent against SARS-CoV-2. Gilteritinib inhibits Axl and consequently downregulates the p38/MAPK pathway, which is involved in proinflammatory cytokine production.	[87]
In vitro study	Identification of bemcentinib as an antiviral agent against SARS-CoV-2. Bemcentinib inhibits Axl, which has been observed to be upregulated in COVID-19-infected lung cells. As Axl-mediated signaling is known to downregulate interferon-related host immune responses, its pharmacological inhibition could help in reducing viral infection.	[88]
Clinical trial	Overview of an ongoing clinical trial aimed to evaluate different drugs, including bemcentinib, as candidate agents for COVID-19 treatment.	[89]
Case report	Case report showing the successful use of gilteritinib in a patient with FLT3-mutated acute myeloid leukemia and severe COVID-19.	[90]
In vitro study	Identification of Axl as a candidate pharmacological target to revert SARS-CoV-2-induced epithelial-to-mesenchymal transition (EMT). Axl is a tyrosine kinase receptor typical of a mesenchymal phenotype, the expression of which is induced by SARS-CoV-2 infection and drives the EMT responsible for ARDS. The authors hypothesize that Axl inhibition by gilteritinib and bemcentinib, two drugs with proven antiviral activity, will not only reduce viral infection load but also will improve patients’ conditions by reverting EMT.	[91]

**Table 2:** Summary of the most relevant reviews regarding the involvement of the Gas6 and TAM receptors in SARS-CoV-2 infection and COVID-19 progression and management.

Article Type	Main Findings	Reference
Review	Description of the possible Gas6/TAM axis involvement in SARS-CoV-2 infection and COVID-19 complications. Overview of the first studies focused on TAM-targeted inhibition for COVID-19 management. TAM (in particular Axl) signaling is supposed to be involved at different stages of COVID-19 evolution. In particular, it has been supposed that the TAM pathway supports viral entry but also the development of immunothrombosis, which has been described to be associated with respiratory failure. According to Axl’s supposed role in the viral infection process, the already clinically available Axl inhibitors are being tested in clinical trials as anti-COVID-19 drugs.	[92]
Review	Overview of Axl involvement in SARS-CoV-2 infection. Axl has been described as an alternative receptor for SARS-CoV-2 viral entry. Interestingly, the interaction involves the spike protein N-terminal domain instead of the receptor binding domain that is recognized by ACE-2. Axl’s role as an entry receptor appears of particular interest in those cells and tissues where it is not co-expressed with ACE-2.	[93]
Review	Overview of Axl inhibitors as potential pharmacological treatments for COVID-19. Axl receptor acts as an alternative receptor for SARS-CoV-2 entry and its pharmacological inhibitors are currently being tested as potential anti-COVID-19 drugs.	[94]
Review	Overview of Axl inhibitors (gilteritinib and bemcentinib) as antiviral agents against COVID-19. Gilteritinib and bemcentinib antiviral action mainly rely on their ability to inhibit Axl signaling and consequently the downstream p38/MAPK pathway.	[95]

## Data Availability

Not applicable.
